# Effect of high‐amylose corn starch addition on canning of yellow alkaline noodle composed of wheat flour and microbial transglutaminase: Optimization by RSM

**DOI:** 10.1002/fsn3.667

**Published:** 2018-05-03

**Authors:** Lida Shahsavani Mojarrad, Ali Rafe

**Affiliations:** ^1^ Department of Food Processing Research Institute of Food Science and Technology (RIFST) Mashhad Iran

**Keywords:** cooking loss, noodle, resistant starch, response surface methodology, texture

## Abstract

Mixture experiment was applied to optimize the canned cross‐linked high‐amylose starch yellow alkaline noodle formula. Processing factors of canned yellow alkaline noodle (YAN) cross‐linked by MTGase at different levels of Hylon VII were surveyed. The factors were wheat flour, Hylon VII starch (HAS) as a high‐amylose starch, and water in the mixture to achieve reduction in cooking loss, water uptake, swelling index, and thickness. Due to the effect of retort processing on YAN, pH, color, and texture were also examined. Analysis of variance showed that the linear mixture had a significant effect on the noodle properties. Optimum conditions were as follows: wheat flour of 75 g, HAS of 15 g, and water 67 ml. Results showed the suitable effect of Hylon in improving texture of canned YAN as well as high gel strength, neutral pH, lowest cooking loss, water uptake, swelling index, and color after retort processing. Therefore, high‐amylose decreased swelling of starch, cooking yield, and improved hardness which obtain a strong gel and better stability in canned noodle. The pH fall and the attainment of yellow color in the YAN containing Hylon can be explained by the availability of two important amino acids, lysine and glutamine which were involved in both cross‐linking reactions. The synergistic effect of low amount of flour and Hylon produced more tensile and hardness properties in canned noodle. Overall, the canned YAN prepared by adding Hylon developed the stronger gel which can withstand at high thermal retort processing and finally improve the shelf life of the final product.

## 1 INTRODUCTION

Noodle is an appealing staple food consumed extensively in Asian countries with broad variety of products. Yellow alkaline noodle (YAN) is a popular noodle made from flour, water, alkaline salts, and sodium chloride (Hatcher & Anderson, 2007). The alkaline salts are exploited as NaoH, Na_2_Co_3_/K_2_Co_3_ which impart the unique attributes to the Chinese noodle such as attractive appearance, brightness, delightful taste, amazing flavor, and textural properties (Morris, Jeffers, & Engle, 2000; Shiau & Yeh, 2001). Its color is the result of the reaction between the flavones and alkaline water. As the shelf life of YAN is restricted to a few days due to the high water and nutrient contents, many attempts have been performed to improve its shelf life by freezing, drying, frying, chemical preservatives such as potassium sorbate, sodium dehydroacetate, and calcium propionate (Li, Zhu, Guo, Peng, & Zhou, 2011) and organic acids prior to heating and cooling (Fu, 2008). Even though the noodles achieved long shelf life of 1 to 2 years, the health awareness concerning about chemical preservatives and undesirable taste of acidified noodles as well as deficient cooking of instant noodle made a soft and sticky surface reduced the desirability among the consumers. Therefore, the most attractive noodle is still the fresh noodle due to its unique flavor and taste. As a result, lack of proper procedure in extending shelf life of noodle without any undesirable changes is completely perceivable.

Thermal processing is a conventional technique to extend shelf life of foods with the least effect on the nutrient content without involving any chemical preservatives. Although high heat processing destroys microorganisms and extends the shelf life, it damages some sensorial properties, particularly texture, as well as nutritional quality such as vitamins (Awuah, Ramaswamy, & Economides, 2007; Gokhale & Lele, 2014). As the structure of noodle is comprised of starch, it is very delicate to harsh conditions in thermal processing and the overall structure may collapse. Furthermore, the noodle texture is soft and soggy and can be crumbled into small pieces. Accordingly, the main challenge in thermal processing of YAN is to retain the gel structure of the processed noodle. Moreover, the firm, elastic, and smooth texture of YAN is preferred by the most consumers (Fu, 2008).

As amylose plays a critical role in gel forming and gel structure, resistant starches (RS) such as high‐amylose corn starches (>70% amylose) received much attention for its health benefits and functional properties (Shahsavani Mojarrad & Rafe, 2017; Shahsavani Mojarrad, Rafe, Sadeghian, & Niazmand, 2017). It has been found that high amylose improves the gel strength and can endure high heat treatments such as retort processing owning to their linear structure (Shahsavani Mojarrad & Rafe, 2017; Shahsavani Mojarrad, Rafe, Sadeghian, et al., 2017; Yeoh, Alkarkhi, & Easa, 2013). Therefore, it was partially used to substitute wheat flour to improve the quality and texture of YAN. Besides, the food‐based protein cross‐linking agents like microbial transglutaminase (MTGase) and soy protein isolate (SPI) were applied to develop the gel structure of the starchy products (Gan, Ong, Wong, & Easa, 2009; Shahsavani Mojarrad, Rafe, Sadeghian, et al., 2017; Yeoh, Alkarkhi, Ramli, & Easa, 2011). SPI addition to YAN not only promotes the nutritional quality and community health but also enhances the textural characteristics of YAN (Dube, Schäfer, Neidhart, & Carle, 2007; Foo, Yew, Liong, & Easa, 2011; Gerrard, 2002; Jiang & Zhao, 2010; Kuraishi, Yamazaki, & Susa, 2001). Furthermore, MTGase has shown the protective effect on the lysine residue from various deteriorative reactions such as Millard reaction and improve the quality of YAN (Choy, Hughes, & Small, 2010; Gan et al., 2009; Seguro, Nio, & Motoki, 1996; Shahsavani Mojarrad, Rafe, Sadeghian, et al., 2017; Wu & Corke, 2005).

The combination of MTGase and SPI has shown the improvement of mechanical and microstructure properties of YAN (Gan et al., 2009; Shahsavani Mojarrad, Rafe, Sadeghian, et al., 2017). However, the combinative application of them with high‐amylose corn starch as a resistant starch in canned YAN during typical food processing (*F*_0_~5 min) has not been investigated. Thereby, the aim of the current research was to utilize crossed mixture design to optimize the formulation of the mixture including Hylon VII, wheat flour, and water to achieve the high gel strength, neutral pH, lowest cooking loss, water uptake, swelling index, thickness, and suitable color of YAN after retort processing.

## 2 MATERIALS AND METHODS

### 2.1 Materials

Basic ingredients for yellow alkaline noodle preparation (wheat flour and alkaline salt) were purchased from the local supermarket (Penang, Malaysia). High‐amylose corn starch (Hylon VII) was obtained from Ingredion (10 Finderne Avenue Bridgewater, New Jersey, USA). Microbial transglutaminase‐K (activity: 4 units/100 mg) was purchased from Ajinomoto Co., Inc. (Tokyo, Japan). A commercial grade of soy protein isolate (SPI) with 90% protein content was obtained from Sim Company (Sdn. Bhd., Penang, Malaysia). All of the other reagents used in this work were supplied from Sigma‐Aldrich Company (Selangor, Malaysia).

### 2.2 Noodle preparation

Yellow alkaline noodle formulations at different levels of Hylon VII are determined by design expert and given in Table 1. In brief, alkaline salt (9:1 sodium and potassium carbonate) was dissolved in distilled water (1% w/w) and added to appropriate amount of wheat flour/Hylon VII. Other ingredients such as cross‐linking agent (MTGase) and SPI were incorporated by a mixer (Kitchen Aid, Benton Harbor, MI, USA). Then, the mixture was incorporated at speed 1 and it was raised up every level for each subsequent minute until it reached speed 6. The speed of the mixer was gradually slowed down and stopped at the 10th minute. The dough was removed and placed in a plastic bag for sheeting with a pasta machine (Shule, Changzhou, Jiangsu, China) with an initial gap setting and width 6 that corresponds to approximately 2 mm. The dough was passed over several roller gaps to reach the desired thickness using the noodle machine with the noodle sheet being folded between passes to ensure homogeneity.

**Table 1:** Experimental design (D‐optimal) for yellow alkaline noodle formulation with actual levels^a^

Run	Ingredients			Observed response^b^					
	Flour (g)	Hylon VII (g)	Water (g)	Cooking loss (%)	Water uptake (g)	Swelling index	Thickness (mm)	Hardness (N)	Tensile strength (Pa)
A	90	10	60	NA	NA	NA	NA	NA	NA
B	85	12.5	62.5	7.39	164.69	4.63	0.8	2.81	31.22
C	80	10	70	5.79	108.9	3.38	1.4	3.01	34.8
D	80	15	65	6.13	139.02	4.03	1	4.29	29.32
E	85	15	60	NA	NA	NA	NA	NA	NA
F	75	15	70	7.03	140.83	4.07	1.3	4.31	29.01
G	90	10	60	NA	NA	NA	NA	NA	NA
H	78.75	13.75	67.5	7.3	157.86	4.49	0.8	4.0	30.10
I	87.5	12.5	60	NA	NA	NA	NA	NA	NA
J	82.5	12.5	65	7.14	159.75	4.54	0.8	3.82	31.3
K	85	15	60	NA	NA	NA	NA	NA	NA
L	80	10	70	7.86	135.03	3.97	1.4	2.90	34.1
M	75	15	70	6.18	137.37	4.01	1.3	4.38	29.01
N	85	10	65	6.64	147.18	4.25	1.1	2.29	34.41

a The amount of SPI, salt, alkaline salt, and MTGase were 5%, 1%, 1%, and 0.5% in all of the formula, respectively.

b NA means at this level of ingredients, the dough did not form and produce the noodle.

The same machine was used in slitting for the noodle piece to a flat, rectangular shape of the noodles. Noodles were slit in such a way that they would not be broken easily during tensile analysis. They were coated with a thin layer of flour to avoid from sticking. Then, they were incubated at 40°C for 5 hr (the optimal temperature for MTGase) followed by steaming using a domestic steamer for 30 min. Ultimately, the noodles were cooled down to ambient temperature under the fan after steaming (Yeoh et al., 2013).

### 2.3 Preparation of noodles via retort processing

Approximately 60 g of noodles was filled into aluminum can (300 × 407 imperial size, 3 by 4 07/16 inches or 73 × 113 mm) and filled with distilled water to leave a headspace of ~7 mm. The noodle cans were exhausted at about 75°C for 10 min in a steam chamber and seamed by a can seamer (Metal Box No. 1‐A Double seamer, London, UK). The cans were retorted in a laboratory scale autoclave (Hirayama HA‐240M; Kasukabe‐shi, Saitama, Japan) at 121°C for 30 min to achieve *F*_*0*_ value ~5. *F*_0_ is the equivalent exposure time at 121°C of the actual exposure time at a variable temperature, calculated for an ideal microorganism with a temperature coefficient of destruction equal to 10°C. After retort processing, the cans were cooled down immediately under flowing tap water and stored at ambient temperature (~30°C) for 3 weeks prior to analysis.

### 2.4 Retort cooking loss

It was determined by evaporating the can's liquid to dryness overnight in a conventional oven at 100°C according to the AACC method 66‐50 (AACC, 2000). Retort cooking loss (%) was calculated as follows (Khouryieh, Herald, & Aramouni, 2006):

(1) $$\begin{array}{cc} \text{Retort}\; & \text{cooking}\;\text{loss}\;(\%)\\ & =\frac{\text{Dried}\;\text{residue}\;\text{in}\;can^{\prime }s\;\text{liquid}\left(g\right)}{\text{Noodle}\;\text{weight}\;\text{before}\;\text{retort}\;\text{cooking}\left(g\right)}\times 100 \end{array}$$

### 2.5 Water uptake

Noodle hydration was measured as the difference between noodles weight after and before retort cooking according to the method of Khouryieh et al. (2006) and Yeoh et al. (2013).

### 2.6 Noodle thickness

The noodle thickness was measured using a manual micrometer (Dial Thickness Gauge Mitutoyo MI 7305, Takarsu‐ku, Kawasaki, Kanagawa, Japan). The increment thickness was calculated as follows:

(2) $$\text{Increment}\;\text{thickness}=\frac{T_{i}\left(mm\right)}{T_{f}\left(mm\right)}\times 100$$

where *T*_*i*_ and *T*_*f*_ are the noodle thickness prior and after retort processing, respectively.

### 2.7 Noodle swelling index

It was determined by the following equation:

(3) $$\begin{array}{cc} \mathrm{Swelling}\;\mathrm{index}= & \text{(weight}\;\;\text{after}\;\;\text{retort}\;\;\text{cooking(g))/}\\ & \text{(weight}\;\;\text{before}\;\;\text{retort}\;\;\text{cooking (g))} \end{array}$$

### 2.8 pH measurement of noodles and liquid

The noodles (10 g) were homogenized with 100 ml deionized water for 5 min and allowed to stand for 30 min. The homogenized suspension was filtered. The pH of the filtrate and can's liquid was measured using pH meter (Delta 320; Mettler‐Toledo Instrument Co., Ltd., Shanghai, China).

### 2.9 Color analysis

Color analysis of noodles was carried out using a colorimeter (Model Minota CM‐3500d; Konica Minolta, Co., Ramsey, NJ, USA) equipped with D65 illuminant using the CIE 1976 L*, a* and b* color scale.

### 2.10 Texture profile analysis and tensile strength

Texture profile analysis (TPA) of noodles was determined using a texture analyzer (TA‐TX2 model; Stable Micro Systems, Surrey, UK) fitted with a 5‐kg load cell as described by Choy et al. (2010). Measurements were taken at room temperature (~22°C). The calibration settings were the 5‐kg load cell with a return trigger path at 15 mm. The measurement mode settings of compression (pretest; test and post‐test) were set to a speed of 2.0 mm/s; strain at 75%, trigger type at auto 10 g, and 35‐mm cylinder probe were used. Five strands of noodles were positioned straight and flat adjacently to one another on the platform securely lined with filter paper fastened by double‐sided adhesive tape. From force–time curves of the TPA, hardness was determined (Park, Hong, & Baik, 2003).

Tensile strength of noodles was determined using the Texture Analyzer fitted with a 2.5‐kg load cell (Gan et al., 2009). Before starting the analysis, rig calibration was performed. The distance the probe should move apart was set to be 15 mm. The settings of the instrument were as follows: mode: measure force in tension; option: return to start; pretest speed: 3.0 mm/s; test speed: 3.0 mm/s; post‐test speed; 5.0 mm/s; distance: 100 mm. Ten strands of noodles from each treatment were cooked, cooled, and drained and stored for 10 min at 25°C (Kruger, Anderson, & Dexter, 1994). The noodles were then tested individually by placing one end into the lower rig arm slot and winding the loosened arm sufficiently, in order to anchor the noodle end. The arm was tightened, and the same procedure was performed to anchor the other noodle end to the upper arm. From the force–displacement curve, the maximum slope was recorded and tensile strength was calculated as σ *= F/A,* where σ is the tensile strength (Pa), *F* represents the maximum load or peak force (N), and *A* represents the cross‐sectional area of the noodle strand (m^2^).

### 2.11 Experimental design

In mixture experiment, the variables are dependent and changing the level of one variable will alter the level of at least one other variable in the experiment. As the sum of the variables must always be 1.0% or 100%, experimentation is subject to the following constraint:

(4) $$\sum_{i=1}^{q}X_{i}=1$$

where *q* is the number of variables (ingredients) in the mixture, and *X* represents the proportion of the *i*th ingredient in the mixture.

Different mathematical models are utilized to analyze data in the mixture experiment as suggested by Scheffe's model (Cornell, 2002). The canonical or Scheffe's models are as follows (Bello & Castro Vierira, 2011):

(5) $$\text{Linear}:Q\left(y\right)=\sum_{i=1}^{q}\beta_{i}X_{i}$$

(6) $$\text{Quadratic}:Q\left(y\right)=\sum_{i=1}^{q}\beta_{i}X_{i}+\sum_{1\leq i<j}^{q}\beta_{ij\;\;}X_{i}X_{j}$$

(7) $$\text{Special}\;\text{cubic}:Q\left(y\right)=\sum_{i=1}^{q}\beta_{i}X_{i}+\sum_{1\leq i<j}^{q}\beta_{ij\;\;}X_{i}X_{j}+\sum_{1\leq i<j<k}^{q}\beta_{ij\;}X_{i}X_{j}X_{k}$$

(8) $$\begin{array}{cc} \text{Full}\;\text{cubic}:Q\left(y\right)= & \;\sum_{i=1}^{q}\beta_{i}X_{i}+\sum_{1\leq i<j}^{q}\beta_{ij\;\;}X_{i}X_{j}+\sum \gamma_{ij\;}X_{i}X_{j}\left(\;X_{i}-X_{j}\right)\\ & +\sum_{1\leq i<j<k}^{}\beta_{ij\;}X_{i}X_{j}X_{k} \end{array}$$

where *Q(y)* is the expected response, *q* is the total number of components in the mixture, β_*i*_ is the expected response to the pure mixture (linear blending), β_*ij*_ is the access response from the quadratic β_*ij*_ *X*_*i*_ *X*_*J,*_ term over the linear model, β_*ijk*_ is the ternary blending among the three components in the interior of the mixture, and γ_ij_ represents both synergistic and antagonistic blending along the *x*_*i*_ *–x*_*j*_ edge.

If the process variables such as time and temperature are included in the model, the quadratic model in the mixture components can be written as:

(9) $$Q\left(y\right)=\sum_{i=1}^{q}\beta_{i}X_{i}+\sum_{1\leq i<j}^{q}\beta_{ij\;\;}X_{i}X_{j}+\sum_{k=1}^{r}\left[\sum_{i=1}^{q}\alpha_{\mathrm{ik}}X_{i}+\sum \sum_{1<i<j}^{q}\alpha_{\mathrm{ik}}X_{i}X_{j}\right]Z_{k}$$

where *r*, α_*ik*_, α_ijk_, and *Z*_k_ represent the number of process variables, the effect of process variable on the linear blending, the effect of process variable on the binary blending of the mixture and the process variables, respectively.

D‐optimal design was used to run the experiment due to minimizing of generalized variance of the parameters of the model and the volume of the confidence ellipsoid for the unknown parameters. Therefore, D‐optimal design was carried out to study the effect of wheat flour (*X*_1_ = 75–90 g), and Hylon VII (*X*_2_ = 10–15 g) on the retorted noodle and adjusting water (*X*_3_ = 60–70 ml) were used accordingly.

### 2.12 Statistical analysis

Collected data were processed using a commercial statistical package, Design Expert version 7.1.6 (Stat‐Ease Inc; Minneapolis, MN, USA). The software was used for the analysis of variance (ANOVA), mathematical modeling, regression analysis, and optimization. The response surface plots were generated for different interactions. The contour plots for all responses were superimposed and regions that best satisfy all the constraints were selected as optimum conditions. The noodle properties including water absorption, increment thickness, width, pH, color, and TPA parameters were evaluated in triplicates, and data were averaged. Data were presented in mean ± standard deviation (*SD*). The Duncan's multiple range test at 5% level was applied to evaluate significant differences between the means of each treatment.

## 3 RESULTS AND DISCUSSION

### 3.1 Effect of retort processing on cooking loss and water uptake

The experimental range for each ingredient was selected based on the preliminary experiments. Wheat flour (A) varied from 75 to 90 g, and Hylon VII (B) was between 10 and 15 g. Six responses including cooking loss, water uptake, swelling index, thickness, hardness, and tensile strength were surveyed. The results of 14 runs using D‐optimal design are provided in Table 1. The results of fitting and quadratic models are also given in Table 2. The results observed for different composition were 5.79% to 7.86% for cooking loss, 0.8 to 1.4 mm for thickness, 108.9 to 159.75 g for water uptake, and 3.38 to 4.63 for swelling index. The results of analysis of variance (ANOVA) for ingredients indicated that the contribution of ingredients was significant for cooking loss, water uptake, swelling index, and thickness at *p* < 0.05. All responses were fitted using the quadratic model for all responses, and the fitted quadratic models for cooking loss, water uptake, swelling index, and thickness in actual values are given in Eqs. (10), (11), (12), (13), respectively.

**Table 2:** ANOVA in mixture quadratic model for cooking loss, water uptake, swelling index, and thickness

Source^a^	Sum of square	*d*_f_	Mean square	*F* value	Probe > *F*
Cooking loss, %					
Model	143.98	5	28.80	23.81	0.0001
Linear mixture	110.00	2	55.00	45.48	<0.0001
AB	2.22	1	2.22	1.83	0.2129
AC	24.14	1	24.14	19.96	0.0021
BC	7.63	1	7.63	6.31	0.0362
Residual	9.67	8	1.21		
Total	153.66	13			
Water uptake, g					
Model	68,019.28	9	7,557.70	87.03	0.0003
Linear mixture	61,918.16	2	30,959.08	356.49	<0.0001
AB	487.07	1	487.07	5.61	0.0770
AC	1,131.99	1	1,131.99	13.03	0.0225
BC	433.45	1	433.45	4.99	0.0892
ABC	322.30	1	322.30	3.71	0.1263
AB(A–B)	486.37	1	486.37	5.60	0.0771
AC(A–C)	2,775.38	1	2,775.38	31.96	0.0048
BC(B–C)	464.56	1	464.56	5.35	0.0818
Residual	347.37	4	86.84		
Total	68,366.65	13			
Swelling index					
Model	56.42	9	6.27	142.58	0.0001
Linear mixture	51.82	2	25.91	589.41	<0.0001
AB	0.36	1	0.36	8.11	0.0465
AC	0.86	1	0.86	19.45	0.0116
BC	0.32	1	0.32	7.22	0.0548
ABC	0.24	1	0.24	5.36	0.0816
AB(A–B)	0.36	1	0.36	8.10	0.0466
AC(A–C)	2.12	1	2.12	48.27	0.0023
BC(B–C)	0.35	1	0.35	7.92	0.0481
Residual	0.18	4	0.044		
Total	56.59	13			
Thickness, mm					
Model	4.43	9	0.49	6.366 × 10^7^	<0.0001
Linear mixture	4.32	2	2.16	6.366 × 10^7^	<0.0001
AB	0.00312	1	0.00312	6.366 × 10^7^	<0.0001
AC	0.025	1	0.0025	6.366 × 10^7^	<0.0001
BC	0.00287	1	0.00287	6.366 × 10^7^	<0.0001
ABC	0.00251	1	0.00251	6.366 × 10^7^	<0.0001
AB(A–B)	0.00312	1	0.00312	6.366 × 10^7^	<0.0001
AC(A–C)	0.061	1	0.061	6.366 × 10^7^	<0.0001
BC(B–C)	0.00709	1	0.00709	6.366 × 10^7^	<0.0001
Residual	0.00071	4	0.00071		
Total	4.43	13			

a A: wheat flour, B: Hylon VII, C: water.

(10) $$\begin{array}{cc} \text{Cooking}\;\text{loss}= & -3.95A-26.37B-7.24C+0.17\text{AB}+0.13\text{AC}\\ & +0.29\text{BC} \end{array}$$

(11) $$\begin{array}{cc} \text{Water}\;\text{uptake}= & \;-2381.66A-1.62B+7233.22C+1631.55\text{AB}\\ & -57.95\text{AC}+1537.94\text{BC}-0.6211\text{ABC}\\ & -4.10\text{AB}(A-B)+0.69\text{AC}(A-C)+4.47\text{BC}(B-C) \end{array}$$

(12) $$\begin{array}{cc} \text{Swelling}\;\text{index}= & -65.945A-4409.47B+200.36C+44.25\text{AB}\\ & +1.60\text{AC}+41.78\text{BC}-0.17\text{ABC}-0.11\text{AB}(A-B)\\ & +0.019\text{AC}(A-C)+0.12\text{BC}(B-C) \end{array}$$

(13) $$\begin{array}{cc} \text{Thickness}= & -11.94A-492.23B+34.88C+4.69\text{AB}-0.27\text{AC}\\ & +4.83\text{BC}-0.018\text{ABC}-0.010\text{AB}(A-B)+3.26\text{AC}(A-C)\\ & +0.017\text{BC}(B-C) \end{array}$$

Interaction coefficients generally indicate the synergism and may be used in optimizing the response. The nonzero parameters assist to describe the effects of Hylon VII starch among the process variables. For instance, the coefficient 0.16 for AB and 0.29 for BC in Eq. (10) explains that each variable influences in some way in cooking loss. In general, a positive sign in the fitted models shows the ability of the variables to increase the response and vice versa for the negative sign. AC and BC in all samples showed the interaction of A and C or B and C will be more effective in the canned noodle. However, the AB coefficient is significant negative, indicating that A and B may produce antagonistic effects on reducing cooking loss, water uptake, swelling index, and thickness. In addition, the interactive effect of wheat flour and water (AC) is positive significant effect on cooking loss, water uptake, swelling index, and thickness. Hylon VII starch and water incorporation in cooking loss, swelling index, and thickness have positive significant effect, while the water uptake has a negative significant effect (*p* < 0.05). The mixing of A, B, and C has also negative effect on the swelling index and water uptake.

**Figure 1:**
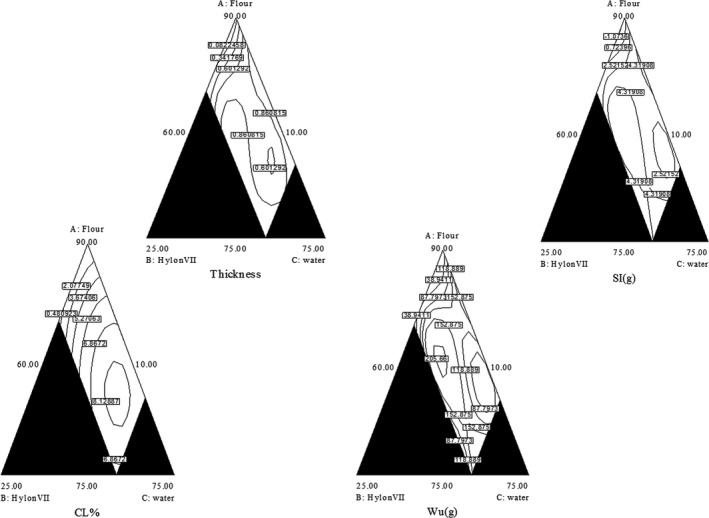
Mixture experiment for cooking loss, water uptake, swelling index, and thickness, A: wheat flour, B: Hylon VII starch, and C: water.

The mixture quadratic models for cooking loss, water uptake, swelling index, and thickness are provided in Figure 1. As the less cooking loss is so vital in noodle processing, the optimum of wheat flour, Hylon, and water is critical. It can be observed that Hylon VII has positive effect on cooking loss. As the amount of Hylon was increased, the cooking loss decreased. As it is a measure of the amount of dry matter loss into the noodle cooking water, it can be used to predict noodle cooking quality. It has been reported that the cooking loss should not be more than 7% in YAN (Kasemsuwan, Bailey, & Jane, 1998; Vijayakumar & Boopathy, 2014). However, it can be affected by the type and amount of noodle ingredients.

Wheat flour showed a positive effect on the water uptake (Figure 1). By increasing the amount of water, the water uptake, thickness, and swelling index were improved. Thus, as the level of Hylon VII starch was increased, the quantity of wheat flour becomes less and water plays an important role in the optimization. As the firm and elastic texture is suitable for YAN, the less swelling index and water uptake are suitable in alkaline noodle hardness. Furthermore, water uptake is a required factor to predict the quality of noodles. Therefore, the less water absorbed by noodles would produce stronger texture of noodles. It was mentioned that increasing the level of amylose would decrease the amount of water uptake. On the other hand, the proper water uptake of noodles should be at least twice their weight after cooking (Kasemsuwan et al., 1998; Ki̇bar, Gönenç, & Us, 2010; Van Hung, Maeda, & Morita, 2006).

Swelling of noodle occurs during cooking in which starch granules hydrate and the water absorbed which brings noodle to swell. Noodle water absorption decreased with amount of the amylose. Low swelling index is desirable for YAN. Extra swollen granules produce a soft bite as increased water uptake of noodle. It has also been found that increasing amylose content is associated by decreasing swelling index of starch–water suspensions (Aravind, Sissons, Fellows, Blazek, & Gilbert, 2012; Maningat & Seib, 2010; Martin et al., 2004; Vijayakumar & Boopathy, 2014). Unsuitable swelling usually results in too sticky and soft texture while using starch with higher level of amylose would cause lesser spontaneous swelling (Tan, Li, & Tan, 2009). Increased amylose content decreased swelling of starch, cooking yield, and hardness. Therefore, high‐amylose starch could be prepared into a strong gel with better stability in canned noodle.

### 3.2 Effect of retort processing on pH and color

The pH values of YAN and its liquid after retort processing were measured, and the results are provided in Table 3. The pH of alkaline noodles depends on salts used, and it is typically within pH 9 to 11 (Fu, 2008; Yeoh et al., 2011). The pH values varied from 7.32 to 8.25, which were lower than the typical range of commercial YAN. It has been revealed that pH fall in canned noodle is due to the formation of acidic Maillard reaction products during heating in the presence of reducing sugars (Gan et al., 2009; Hill et al., 1992). Moreover, the pH of noodles was more than liquid, which may be attributed to less cooking loss during retort processing. In addition, the lowest pH value was seen for the formula containing 15% Hylon VII and 80% wheat flour at 65 ml water. As it can be seen, there was not any statistical significant difference among different formula of YAN (*p* < 0.05). It may be considered owning to the Hylon addition in the YAN formula, which prevents leaching from noodle and loss of the alkaline salts into the boiling water.

**Table 3:** pH and color parameters of processed noodles at various formulation†

Noodle formulation	pH	pH liquid	Color		
			L*	a*	b*
A	NA	NA	NA	NA	NA
B	8.19^a^ ± 0.2	7.87^a^ ± 0.3	45.35^e^ ± 0.23	−2.32^a^ ± 0.04	2.26^a^ ± 0.20
C	8.14^a^ ± 0.1	6.0^c^ ± 0.1	43.02 ^h^ ± 0.20	−2.35^a^ ± 0.05	1.45^b^ ± 0.03
D	8.25^a^ ± 0.3	6.65^b^ ± 0.2	46.09^d^ ± 0.2	−2.47^a^ ± 0. 2	2.16^a^ ± 0.02
E	NA	NA	NA	NA	NA
F	8.05^a^ ± 0.0	7.2^b^ ± 0.2	48.09^b^ ± 0.26	−2.35^a^ ± 0. 1	2.11^a^ ± 0.02
G	NA	NA	NA	NA	NA
H	8.2^a^ ± 0.2	7.76^a^ ± 0.1	47.1^c^ ± 0.0	−2.32^a^ ± 0.02	2.01^a^ ± 0.09
I	NA	NA	NA	NA	NA
J	8.3^a^ ± 0.1	7.01^b^ ± 0.2	45.05^f^ ± 0.23	−2.33^a^ ± 0. 1	2.01^a^ ± 0.00
K	NA	NA	NA	NA	NA
L	7.82^a^ ± 0.4	8.01^a^ ± 0.3	43.01^h^ ± 0.21	−2.31^a^ ± 0.0	1.25^b^ ± 0.03
M	8.03^a^ ± 0.1	6.6^b^ ± 0.2	49.05^a^ ± 0.26	−2.30^a^ ± 0.01	2.09^a^ ± 0.29
N	8.4^a^ ± 0.0	6.98^b^ ± 0.1	43.7^g^ ± 0.21	−2.34^a^ ± 0. 1	1.65^b^ ± 0.03

†Results are means of three replicates. Mean values having different superscripts within the column are significantly different (*p* < 0.05). L*= lightness; a*= redness–greenness; b*= yellowness–blueness.

Noodle color depends on its type and all noodles require high brightness. Alkaline salts can produce white or yellow noodle color (Hou & Kruk, 1998). As yellowness in YAN formula is a key characteristic for consumer acceptance, the laboratory parameters of YAN formula were determined. According to the results of analysis of variance in Table 2, by adding Hylon to YAN formula, the L* value was significantly increased (*p* < 0.05). The L* value ranged from 43 to 49 which was less than the BSA gels cross‐linked by MTGase (Gan et al., 2009). The white gel formation of YAN might be due to the aggregation of protein via MTGase cross‐linking that coincides with heating and high amylose. Furthermore, by applying high‐amylose starch (HAS) in alkaline noodles formula, the yellowness index (b*) and brightness (L*) were significantly increased (*p* < 0.05). The a* value did not show a positive effect of Hylon addition. Consequently, Hylon addition to YAN formula improved the color as well as ensuring product quality and as a result its acceptance by the consumers. The pH fall and the attainment of yellow color in the YAN containing Hylon can be explained by the availability of two important amino acids, lysine and glutamine which both were involved in cross‐linking reactions (Ashoor & Zent, 1984). As the amylose content level was increased from 0 to 15%, the amount of wheat flour or its protein values was reduced. As the MTGase (0.5%) was constant, the ratio of MTGase to lysine was improved and less lysine is available to cross‐linked by MTGase. Consequently, the less cross‐linked network structures were developed at high level of Hylon VII (Shahsavani Mojarrad, Rafe, Sadeghian, et al., 2017). Therefore, less lysine and glutamine would be present to participate in decolorizing process such as Millard reactions. In fact, increasing the amylose level and the ratio of MTGase to wheat protein had a significant effect on the color of YAN.

### 3.3 Effect of retort processing on texture of alkaline YAN

**Figure 2:**
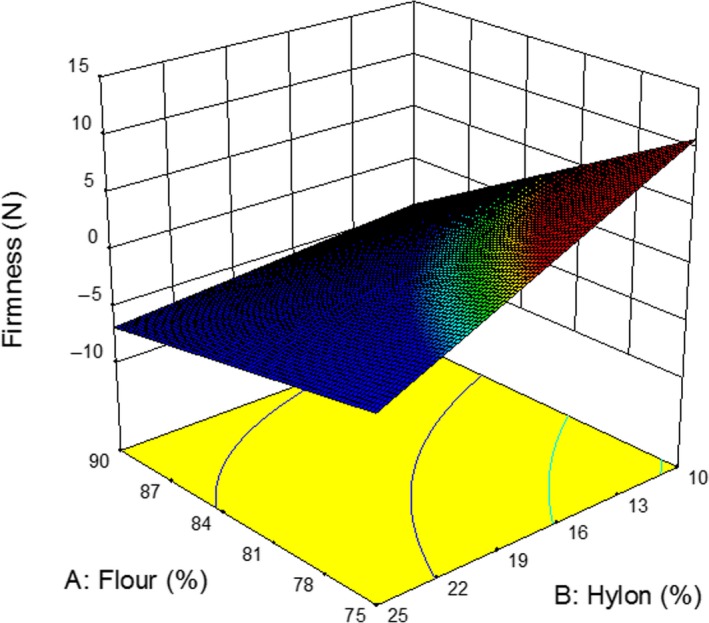
Effect of Hylon addition on the firmness of YAN at water content 67.5%. YAN, yellow alkaline noodle.

**Figure 3:**
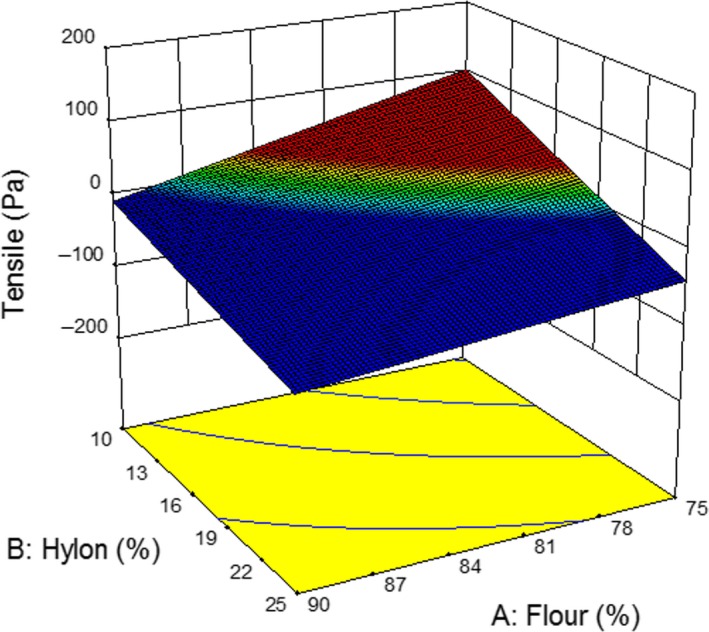
Effect of Hylon addition on the tensile strength of YAN at water content 65.9%. YAN, yellow alkaline noodle.

Tensile strength is an important parameter in determining the quality of the noodles as firm and elastic noodles are highly desirable (Ross, Quail, & Crosbie, 1997; Yeoh et al., 2011). The amount of amylose starch had a positive impact on noodle quality. The results of tensile strength and hardness of the noodles are key factors in determining the quality of the noodles (Table 1). The 3D response for gel hardness and tensile strength is given in Figures 2 and 3, respectively. It is evident that increasing wheat flour and high amylose yielded the less tensile strength. They showed the synergistic effect and low amount of flour and Hylon produced more tensile property. The similar behavior of the ingredients was observed for the hardness (Figure 3). The enhanced gel strength of combined‐cross‐linked gels could be attributed to the formation of denser and finer network that strengthen the protein structure (Gan et al., 2009).

Many small holes are observed on the surface of noodles which could permit the influence of water into the interior of noodle during cooking, and the strand expanded due to the excessive swelling of wheat starch granules. It may be assumed that the HAS granules considered as a filler with ordered structure and filled the holes and cracks in a gel‐like structure (Shahsavani Mojarrad, Rafe, Sadeghian, et al., 2017). Using cross‐linking between amylose and wheat protein, the water uptake was reduced at high temperatures. As the gelatinization degree was increased, the more structured texture of noodle was obtained. At high thermal processing, the amylose was leached from starch granules, but by applying MTGase as cross‐linker, less amylose was leached and thus the texture was preserved. During retrogradation, the gel structure became hard and it is not suitable in this work.

### 3.4 Optimization

Optimization processes provide great opportunities for scientific investigation on food supply development. In general, all responses exhibited a clear peak, suggesting that the optimum conditions for maximum pH, color, and strength are well defined inside the design boundary. The optimum level of independent variables for the formulation of canned noodles with minimum water uptake and cooking loss was 121.16 g and 3.13%, respectively. The acceptable retort cross‐linked high‐amylose yellow alkaline noodle with a good quality of minimum water uptake, cooking loss, swelling index, and thickness was made from the ratio of the combination of three independent variables at 75 g/100 g (A), 15 g/100 g (B), and 67 ml (C). The minimum thickness and swelling index for this value were achieved 0.9% and 3 for retort cross‐linked high‐amylose yellow alkaline noodle.

## 4 CONCLUSION

The cooking loss, water uptake, swelling index, thickness, pH, color, hardness, and tensile strength of canned YAN which cross‐linked by MTGase at different levels of Hylon VII were surveyed. Cooking quality of noodles could be determined by measuring the cooking loss, swelling index, thickness as well as noodle texture. Our findings showed that low swelling index, water uptake, thickness, and cooking loss by applying of Hylon VII. During cooking, starch gelatinization occurs and gelatinized amylose absorbs less water than amylopectin when heated in the presence of water. Due to the more water absorption to interact with gluten, starch, and other components of noodles, overcooked noodles was not produced. In starch noodles, cooking loss during cooking is mostly due to the solubilization of loosely bound gelatinized starch from the surface of the product. As the amount of Hylon was increased, the cooking loss decreased. As it is a measure of the amount of dry matter loss into the noodle cooking water, it can be used to predict noodle cooking quality. Moreover, the water absorption of canned YAN was decreased by adding Hylon. Furthermore, the low swelling index for YAN is desirable. Increased amylose content decreased swelling of starch, cooking yield, and hardness. Therefore, high‐amylose starch could be prepared into a strong gel with better stability in canned noodle. The pH of noodles was more than liquid, which may be attributed to less cooking loss during retort processing. In addition, the lowest pH value was seen for the formula containing 15% Hylon VII and 80% wheat flour at 65 ml of water. Consequently, Hylon addition to YAN formula improved the color as well as ensuring product quality. The pH fall and the attainment of yellow color in the YAN containing Hylon can be explained by the availability of two important amino acids, lysine, and glutamine which were involved in both cross‐linking reactions. The synergistic effect of low amount of flour and Hylon produced more tensile and hardness properties in canned noodle. Overall, the canned YAN prepared by adding Hylon developed the stronger gel which can withstand at high thermal retort processing and finally improve the shelf life of the final product.

## CONFLICT OF INTEREST

The authors declare that they have no conflict of interest and the study does not involve any human or animal testing.

## References

[fsn3667-bib-0001] AACC (2000). Approved method of American Association of Cereal Chemists (10th ed.). St. Paul, MN: AACC.

[fsn3667-bib-0002] Aravind, N. , Sissons, M. , Fellows, C. M. , Blazek, J. , & Gilbert, E. P. (2012). Optimisation of resistant starch II and III levels in durum wheat pasta to reduce in vitro digestibility while maintaining processing and sensory characteristics. Food Chemistry, 136(2), 1100–1109. 23122168 10.1016/j.foodchem.2012.08.035

[fsn3667-bib-0038] Ashoor, S. H. , & Zent, J. B. (1984). Maillard browning of common amino acids and sugars. Journal of Food Science, 49(4), 1206–1207. 10.1111/j.1365-2621.1984.tb10432.x

[fsn3667-bib-0003] Awuah, G. B. , Ramaswamy, H. S. , & Economides, A. (2007). Thermal processing and quality: Principles and overview. Chemical Engineering and Processing, 46, 584–602. 10.1016/j.cep.2006.08.004

[fsn3667-bib-0004] Bello, L. H. A. , & Castro Vierira, A. F. (2011). Tutorial for mixture‐process experiments with an industrial application. Brazilian Operations Research Society, 21(3), 543–546.

[fsn3667-bib-0005] Choy, A. L. , Hughes, J. G. , & Small, D. M. (2010). The effects of microbial transglutaminase, sodium stearoyl lactylate and water on the quality of instant fried noodles. Food Chemistry, 122, 957–964. 10.1016/j.foodchem.2009.10.009

[fsn3667-bib-0006] Cornell, J. A. (2002). Experiments with mixtures: Designs, models and the analysis of mixture data (3rd ed.). New York, NY: John Wiley & Sons.

[fsn3667-bib-0007] Dube, M. , Schäfer, C. , Neidhart, S. , & Carle, R. (2007). Texturisation and modification of vegetable proteins for food applications using microbial transglutaminase. European Food Research and Technology, 225(2), 287–299. 10.1007/s00217-006-0401-2

[fsn3667-bib-0008] Foo, W. T. , Yew, H. S. , Liong, M. T. , & Easa, A. M. (2011). Influence of formulations on textural, mechanical and structural breakdown properties of cooked yellow alkaline noodles. International Food Research Journal, 18, 1295–1301.

[fsn3667-bib-0009] Fu, B. X. (2008). Asian noodles: History, classification, raw materials, and processing. Food Research International, 41(9), 888–902. 10.1016/j.foodres.2007.11.007

[fsn3667-bib-0010] Gan, C. Y. , Ong, W. H. , Wong, L. M. , & Easa, A. M. (2009). Effects of ribose, microbial transglutaminase and soy protein isolate on physical properties and in‐vitro starch digestibility of yellow noodles. LWT – Food Science and Technology, 42(1), 174–179. 10.1016/j.lwt.2008.05.004

[fsn3667-bib-0011] Gerrard, J. A. (2002). Protein–protein crosslinking in food: Methods, consequences, applications. Trends in Food Science & Technology, 13(12), 391–399. 10.1016/S0924-2244(02)00257-1

[fsn3667-bib-0012] Gokhale, S. , & Lele, S. (2014). Retort process modeling for Indian traditional foods. Journal of Food Science and Technology, 51(11), 3134–3143. 10.1007/s13197-012-0844-3 26396305 PMC4571269

[fsn3667-bib-0013] Hatcher, D. W. , & Anderson, M. J. (2007). Influence of alkaline formulation on oriental noodles colour and texture. Journal of Cereal Science, 84, 253–259. 10.1094/CCHEM-84-3-0253

[fsn3667-bib-0039] Hill, W. R. , Weber, S. C. , & Stewart, A. J. (1992). Food limitation of two lotic grazers: quantity, quality and size‐specificity. Journal of the North American Benthological Society, 11(4), 420–432.

[fsn3667-bib-0014] Hou, G. , & Kruk, M. (1998). Asian noodle technology. Technical Bulletin, 20(12), 1–10.

[fsn3667-bib-0015] Jiang, S. J. , & Zhao, X. H. (2010). Transglutaminase‐induced cross‐linking and glucosamine conjugation in soybean protein isolates and its impacts on some functional properties of the products. European Food Research and Technology, 231(5), 679–689. 10.1007/s00217-010-1319-2

[fsn3667-bib-0016] Kasemsuwan, T. , Bailey, T. , & Jane, J. (1998). Preparation of clear noodles with mixtures of tapioca and high‐amylose starches. Carbohydrate Polymers, 36(4), 301–312. 10.1016/S0144-8617(97)00256-7

[fsn3667-bib-0017] Khouryieh, H. , Herald, T. , & Aramouni, F. (2006). Quality and sensory properties of fresh egg noodles formulated with either total or partial replacement of egg substitutes. Journal of Food Science, 71, 433–437. 10.1111/j.1750-3841.2006.00060.x

[fsn3667-bib-0018] Ki̇bar, E. A. A. , Gönenç, I. , & Us, F. (2010). Gelatinization of waxy, normal and high amylose corn starches. GIDA – Journal of Food, 35(4), 237–244.

[fsn3667-bib-0019] Kruger, J. E. , Anderson, M. J. , & Dexter, J. E. (1994). Effect of flour refinement on raw Cantonese noodle color texture. Cereal Chemistry, 71, 177–182.

[fsn3667-bib-0020] Kuraishi, C. , Yamazaki, K. , & Susa, Y. (2001). Transglutaminase: Its utilization in the food industry. Food Reviews International, 17(2), 221–246. 10.1081/FRI-100001258

[fsn3667-bib-0021] Li, M. , Zhu, K. , Guo, X. , Peng, W. , & Zhou, H. (2011). Effect of water activity (*a* _w_) and irradiation on the shelf‐life of fresh noodles. Innovative Food Science and Emerging Technologies, 12, 526–530. 10.1016/j.ifset.2011.06.005

[fsn3667-bib-0023] Maningat, C. C. , & Seib, P. A. (2010). Understanding the physicochemical and functional properties of wheat starch in various foods. Cereal Chemistry, 87(4), 305–314. 10.1094/CCHEM-87-4-0305

[fsn3667-bib-0024] Martin, J. M. , Talbert, L. E. , Habernicht, D. K. , Lanning, S. P. , Sherman, J. D. , Carlson, G. , & Giroux, M. J. (2004). Reduced amylose effects on bread and white salted noodle quality. Cereal Chemistry, 81(2), 188–193. 10.1094/CCHEM.2004.81.2.188

[fsn3667-bib-0025] Morris, C. F. , Jeffers, H. C. , & Engle, D. A. (2000). Effects of processing, formula and measurement variables on alkaline noodles colour toward and optimizes laboratory system. Cereal Chemistry, 77, 77–85. 10.1094/CCHEM.2000.77.1.77

[fsn3667-bib-0026] Park, C. S. , Hong, B. H. , & Baik, B. K. (2003). Protein quality of wheat desirable for making fresh white salted noodles and its influence on processing and texture of noodles. Cereal Chemistry, 80, 297–303. 10.1094/CCHEM.2003.80.3.297

[fsn3667-bib-0027] Ross, A. , Quail, K. , & Crosbie, G. (1997). Physicochemical properties of Australian flours influencing the texture of yellow alkaline noodles. Cereal Chemistry, 74(6), 814–820. 10.1094/CCHEM.1997.74.6.814

[fsn3667-bib-0028] Seguro, K. , Nio, N. , & Motoki, M. (1996). Some characteristics of a microbial protein cross‐linking enzyme: Transglutaminase In Parris N., Kato A., Creamer N. K., & Pearce J. (Eds.), Macromolecular interactions in food technology (pp. 271–280). Columbus, OH: ACS Symposium Series 650.

[fsn3667-bib-0029] Shahsavani Mojarrad, L. , & Rafe, A. (2017). Textual, thermal, and microstructural properties of composite gel of wheat flour/high amylose corn starch: Effect of high temperatures. Iranian Food Science and Technology Research Journal, Article in Press.

[fsn3667-bib-0030] Shahsavani Mojarrad, L. , Rafe, A. , Sadeghian, A. , & Niazmand, R. (2017). Effects of high amylose corn starch and microbial transglutaminase on the textural and microstructural properties of wheat flour composite gels at high temperatures. Journal of Texture Studies, 48(6), 624–632. 10.1111/jtxs.12277 28557021

[fsn3667-bib-0031] Shiau, S. Y. , & Yeh, A. I. (2001). Effects of alkali and acid on dough rheological properties and characteristics of extruded noodles. Journal of Cereal Science, 33, 27–37. 10.1006/jcrs.2000.0344

[fsn3667-bib-0032] Tan, H. Z. , Li, Z. G. , & Tan, B. (2009). Starch noodles: History, classification, materials, processing, structure, nutrition, quality evaluating and improving. Food Research International, 42(5), 551–576. 10.1016/j.foodres.2009.02.015

[fsn3667-bib-0033] Van Hung, P. , Maeda, T. , & Morita, N. (2006). Waxy and high‐amylose wheat starches and flours – Characteristics, functionality and application. Trends in Food Science & Technology, 17(8), 448–456. 10.1016/j.tifs.2005.12.006

[fsn3667-bib-0034] Vijayakumar, T. P. , & Boopathy, P. (2014). Optimization of ingredients for noodle preparation using response surface methodology. Journal of Food Science and Technology, 51(8), 1501–1508. 10.1007/s13197-012-0641-z 25114340 PMC4108670

[fsn3667-bib-0035] Wu, J. , & Corke, H. (2005). Quality of dried white salted noodles affected by microbial transglutaminase. Journal of the Science of Food and Agriculture, 85, 2587–2594. 10.1002/(ISSN)1097-0010

[fsn3667-bib-0036] Yeoh, S. Y. , Alkarkhi, A. F. , & Easa, A. M. (2013). Effect of cross‐linking agents on physicochemical, textural properties and microstructure of canned soy protein isolate‐yellow alkaline noodles prepared by retort processing. Journal of Food Processing and Preservation, 38(3), 1187–1197.

[fsn3667-bib-0037] Yeoh, S. Y. , Alkarkhi, A. F. , Ramli, S. B. , & Easa, A. M. (2011). Effect of cooking on physical and sensory properties of fresh yellow alkaline noodles prepared by partial substitution of wheat flour with soy protein isolate and treated with cross‐linking agents. International Journal of Food Sciences and Nutrition, 62(4), 410–417. 10.3109/09637486.2010.539555 21306189

